# Familial Correlates of Leisure Time Activities among Polish Early School-Age Children: A Cross-Sectional Study

**DOI:** 10.3390/ijerph18073704

**Published:** 2021-04-01

**Authors:** Anna Kawalec, Krystyna Pawlas

**Affiliations:** Department of Hygiene, Wroclaw Medical University, 50-345 Wroclaw, Poland; wl-10@umed.wroc.pl

**Keywords:** school-age children, leisure time, physical activity, screen time, lifestyle

## Abstract

The after-school period may play a critical role in the accumulation of children’s physical activity and sedentary time. The study aimed to characterize familial correlates of early school-age children’s leisure time activities. A cross-sectional study was conducted among a group of 223 children (mean age 8.7 ± 0.5) and their parents. The percentage of children with daily leisure time physical activity (LTPA) >1 h was 23.32%, and with daily screen time <2 h was 32.74%. The average children’s leisure time physical activity was significantly higher on weekend days than on weekdays (114.85 vs. 89.43 min, *p* = 0.005). Similarly, the average screen time was higher on weekend days than on weekdays (95.50 vs. 66.10 min, *p* < 0.001). The multivariate regression analysis revealed that independent predictors of children’s leisure time physical activity were the father’s education level and the father’s occupational status, whereas at least one parent with higher education correlated negatively with children’s longer screen time. The study showed that children’s leisure time activities are associated with parental education and differ significantly between weekdays and weekend days. These findings underline the need for screening for unfavorable health behaviors among early school-age children, and indicate that health promotion programs should be oriented on both parents and children aiming to improve parental health consciousness, reduce screen time and increase physical activity, especially during the weekend.

## 1. Introduction

Nowadays, we observe unfavorable changes in children’s lifestyles, with a shift toward sedentary behaviors and decreased physical activity levels. Physical activity in childhood is one of the major components required for healthy growth. In particular, it improves motor skills and performance and positively affects skeletal health by increasing bone mineral density [1,2]. Moreover, physical activity is an important determinant of cognitive development, such as language learning, attention, memory and academic achievement [1,3,4]. It has a beneficial impact on emotional and mental well-being, helps in stress management and reduces the risks of cardiovascular diseases, diabetes, cancer, becoming overweight and obese, and other chronic diseases [2].

For children, physical activity (PA) includes play, games, sports, transportation, chores, recreation, physical education, or planned exercise, in the context of family, school, and community activities. According to WHO recommendations, children should accumulate at least 60 min of moderate-to-vigorous intensity physical activity daily [5]. The guidelines for limiting screen time in children are inconsistent and differ according to region and age [6]. Excessive screen time has been shown to be negatively associated with the development of physical and cognitive abilities, and positively with overweight or obesity, sleep problems, depression, anxiety, and addiction [7,8,9]. The Polish National Food and Nutrition Institute recommends school-age children reduce their total daily screen time to less than 2 h.

However, a large proportion of children across different European countries do not meet PA recommendations and spend a lot of time being sedentary [10,11]. The subjectively measured data indicate that from 5 to 47% of children meet PA guidelines, whereas objectively measured data range from 0 to 60% [12]. A heterogeneity between countries is observed, with the highest proportion of children meeting the PA recommendations in Finland (41%), Ireland (38%), and Bulgaria (36%), and the lowest in Italy (13%), Denmark (15%), and Greece (16%) [11,12]. In Poland, the estimated prevalence of sufficient physical activity levels among children is 24% and is higher among boys than girls (30% vs. 19%) [13]. According to data from the Childhood Obesity Surveillance Initiative Report, the percentage of Polish 8-year-olds who are physically active in their leisure time for more than 1 h is 55.9% on weekdays and 92.1% on weekend days. The percentage of children whose screen time is less than 2 h daily on school days is 54.6%, but only 14.8% on weekend days [14].

Total physical activity level in children is composed of in school activities and after-school sport participation or unorganized leisure time physical activity. According to recent cross-sectional studies, the majority of children’s total physical activity is accumulated either in free time (41%) or at school (33%) [15]. It is worth noting that physical activity during physical education (PE) lessons has been shown to be relatively low [16,17]. Only a very small percentage of children meet physical activity guidelines during PE lessons [18]. It is estimated that about 33% of the time in PE lessons is spent in moderate-to-vigorous physical activity (MVPA), which contributes to about 13 to 17% of moderate-to-vigorous physical activity of the whole day [19,20]. Whereas activities during free time account for up to one-third of daily moderate-to-vigorous physical activity and more than one-fifth of children’s daily time spent being sedentary [21]. These findings indicate that the after-school period may play a critical role in the accumulation of both children’s physical activity and sedentary time.

Evidence shows that physical activity level decreases with age, and younger children are generally more active than adolescents. The onset of age-related lowering of physical activity and increase in sedentary time seems to become apparent at the age of about 6 to 7 years [22,23]. Schwarzfischer et al. observed a steep decline in the number of children fulfilling current PA recommendations between 8 and 11 years [23]. These findings suggest that the decline of physical activity and increase of sedentary behaviors start in early school-age, emphasizing that this period is a crucial time for healthy lifestyle intervention.

In Poland, the number of studies evaluating leisure time activities among early school-age children is rather limited. Activities during free time may significantly contribute to total physical activity and sedentary time. In early school-age, parental influence on their children’s health behaviors is of major importance. To develop effective health promotion and educational activities which aim to increase children’s leisure time physical activity and reduce screen time, it is important to improve our understanding of how both parents influence their children’s free-time activities. Since scientific evidence points out that early school-age may be a critical period in shaping health behaviors, and there are no studies in the Polish population of this group of children and their parents, this study aimed to identify familial correlates of leisure time activities among Polish early school-age children.

## 2. Materials and Methods

### 2.1. Participants and Settings

**Figure 1:**
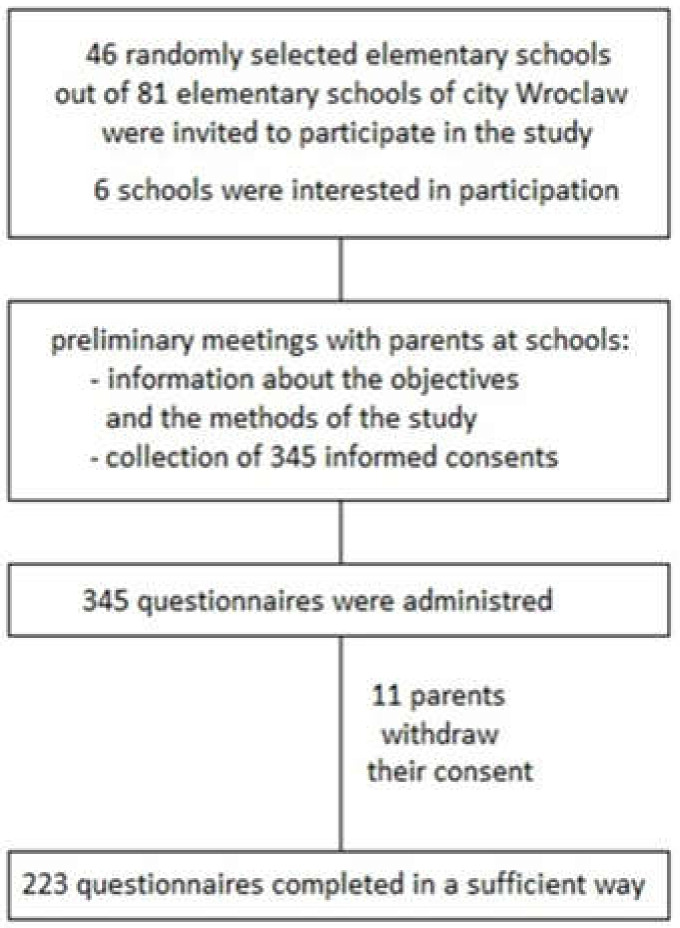
Process of selecting elementary schools and the final study group.

The study was approved by the Bioethics Committee at Wroclaw Medical University and conducted in six elementary schools in the city Wroclaw (Poland) in school years 2017–2018 and 2018–2019. Participation in the study was voluntary. After presenting the aim and methods of this study during preliminary meetings at school, parents were asked to sign an informed consent. The final study group consisted of 223 students of 2nd and 3rd grade and their parents. Figure 1 shows an overview of the process of selecting the schools and the study group formation.

### 2.2. Methods

#### 2.2.1. Information on How Free Time Was Spent by the Child and Selected Familial Correlates

Information about children’s free-time activities and the socio-demographic characteristics of their families was collected with the use of a questionnaire. The original Italian weekly observation diary entitled “Seven days for my health” by Domenico Tiso was designed for the lifestyle assessment of school children aged 6 to 11 years [24]. This diary was translated and adapted for use by Polish children and their parents.

The paper-based questionnaire, in the form of a booklet, was comprised of two separate parts: one for parents and one for children. The questionnaire for parents included questions about age, occupation (full-time, part-time, etc.), education level, and a section assessing their physical activity with the use of the International Physical Activity Questionnaire for Adults (IPAQ). According to the IPAQ scoring, parental physical activity level was classified as high, medium, or low.

The questionnaire for children was designed as a weekly observation diary to be completed by the child, under the supervision of a caregiver. At the end of each day, before bedtime, children answered questions about how they had spent their free time, indicating the type of activity and its duration. The following questions checked if during leisure time the child practiced sport, actively played (running, jumping, dancing, riding a bike, etc.), watched television, played computer games, or used other electronic devices:

- Did you practice sport with a trainer or instructor today? Yes/No
- If yes, how long did the sport classes last?
- Did you play actively today (examples of active play: running, jumping, dancing, riding a bike, etc.)? Yes/No
- If yes, how long did you play for?
- Did you watch television today? Yes/No
- If yes, how long for?
- Did you play computer games, video games, play on a tablet or smartphone today? Yes/No
- If yes, how long for?

#### 2.2.2. The Reliability and Validity of the Child’s Leisure Time Activities Questionnaire

Children reported in their diaries how long they participated in sport classes, played actively, watched television, played on a computer, tablet, smartphone, etc. This questionnaire was previously used in different studies among school-age children in Italy and showed to be adequate for this age group [24].

The assessment of the validity of the child’s leisure time activities questionnaire, using specially designed scales, showed a good psychometric quality, with Cronbach’s alpha = 0.735, standardized Cronbach’s alpha = 0.725, mean correlation between items *r* = 0.166 for the part regarding leisure time physical activity, and Cronbach’s alpha = 0.850, standardized Cronbach’s alpha = 0.858, mean correlation between items *r* = 0.313 for the part focusing on screen time.

### 2.3. Data Analysis

1. For all quantitative features, mean value (M), standard deviation (SD), median (Me), lower (Q1) and upper (Q3) quartiles, and volatility range (Min and Max) were calculated.

2. Significance of differences in mean values of variables (features) with a normal distribution and homogeneous variances were checked with the *t*-Student test.

3. Spearman’s correlation coefficients were generated to examine the unadjusted bivariate associations between the mean daily time of a child’s physical activity and parental physical activity (expressed as MET*min/week), mean time spent by parents on walking or sitting (expressed as min/day).

4. The multivariate regression was performed to investigate the predictors of children’s leisure time physical activity and screen time. The regression model (separate for LTPA and ST) was developed using a set of eight predictors (candidate variables): mother’s and father’s education level (1 = higher education; 2 = vocational; 3 = medium; 4 = gymnasium; 5 = elementary), mother’s and father’s occupational status (1 = full-time; 2 = part-time; 3 = not working), mother’s and father’s physical activity level (3 = high; 2 = medium; 1 = low), at least one parent with higher education, at least one parent with high physical activity level.

5. The significance level was assumed as *p* < 0.05.

6. Analyses were performed using Statistica software (version 13.0 PL; StatSoft Inc., Tulsa, OK, USA; StatSoft, Krakow, Poland).

## 3. Results

### 3.1. Sample Size and Demographic

In total, 223 children participated in this study (124 girls and 99 boys) aged from 7 to 10 years (mean age was 8.7 ± 0.5 years). In this group, 171 questionnaires (76.7%) were completed by the child and both parents, 22 by the child and one parent (9.9%), and 30 (13.4%) by the child only.

### 3.2. The Way of Spending Free Time among Study Group

#### 3.2.1. Complying with the Recommendations for Physical Activity and Screen Time

**Figure 2:**
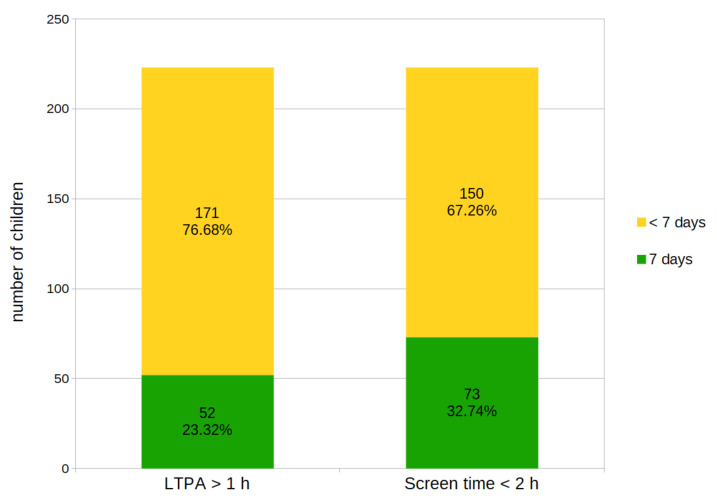
The number of children who complied with the recommendations for physical activity and screen time in their free time.

**Figure 3:**
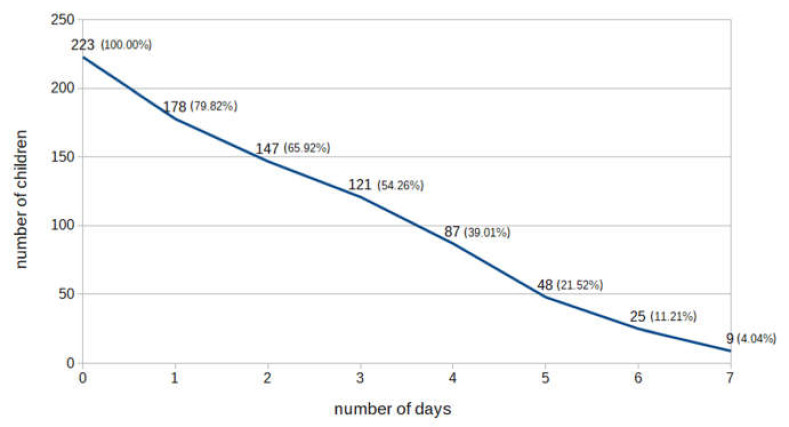
Cumulated number (percentage) of children who, during free time, were both physically active and limited screen time to less than 2 h. The number of children who, each day of the week, were physically active (>1 h) and limited screen time (<2 h) was 9 (4.04%). The number of children who complied with both recommendations at least 6 days per week (6 or 7 days) was 25 (11.21%), etc.

The percentage of children with daily leisure time physical activity (LTPA) above 1 h was 23.32%, and those with daily screen time of less than 2 h was 32.74% (Figure 2). However, only 9 children (4.04%) met these two indicators of a healthy way of spending their free time (Figure 3).

#### 3.2.2. Child’s Leisure Time Physical Activity (LTPA)

**Figure 4:**
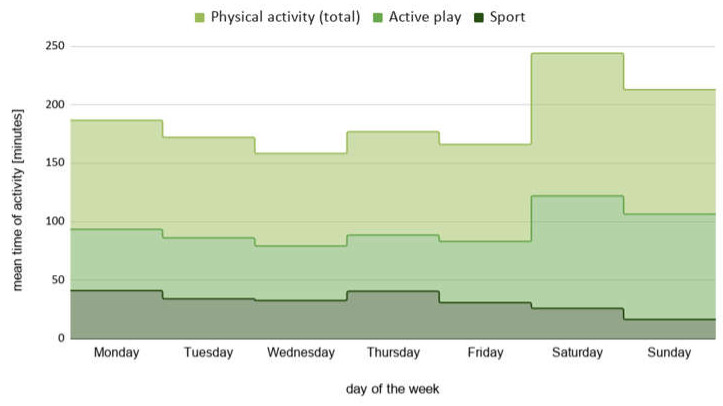
The mean time of total LTPA during the week, with subdivision of sport and active play.

In their free time, children were physically active for approximately 89 min daily during school days and 114 min on weekend days. The mean time of free-time physical activity with subdivision to sport and active play during the whole week is presented in Figure 4. There is a significant difference between leisure time physical activity on school days and weekend days (Table 1).

#### 3.2.3. Child’s Leisure Screen Time

**Figure 5:**
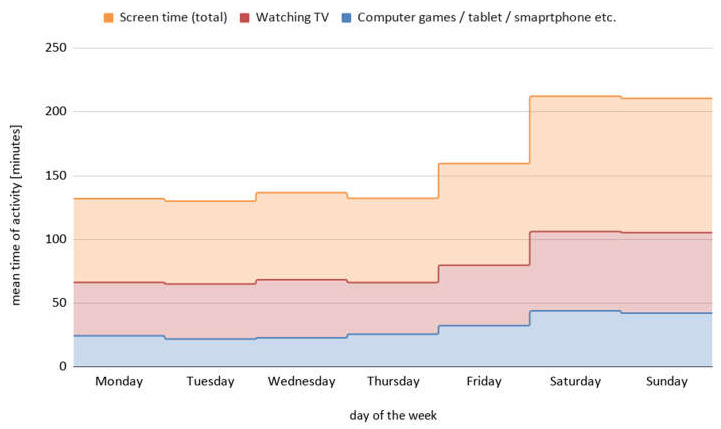
The mean time of leisure screen time during the week, with subdivision to watching television and playing computer games (including the use of a smartphone, tablet, or other electronic devices).

We observed that screen time was approximately 66 min daily during school days, and 95 min on weekend days. Mean time of total leisure screen time, with subdivision to watching television and playing computer games (including the use of a smartphone, tablet, or playing video games), during the whole week is presented in Figure 5. There was a significant difference between screen time on school days and weekend days (Table 2).

According to the recommendations for children, screen time should be limited to less than 2 h per day. In the study group, 73 children (32.74%) met these recommendations, i.e., spent less than 120 min in front of a TV or computer daily. However, screen time lasting more than 2 h each day of the week was observed among 17 children (7.62%).

#### 3.2.4. Child’s Leisure Time Physical Activity and Parental Physical Activity

The analysis of the correlations between the child’s LTPA and parental physical activity level showed a positive correlation between the child’s mean LTPA and the father’s physical activity, as well as the average time spent daily on walking by the mother and father. In contrast, a negative correlation was found between the mean child’s LTPA and the mean daily time spent sedentary by the father. The results are presented in Table 3.

#### 3.2.5. Familial Correlates of Child’s Leisure Time Activities

Multivariate regression was used to examine a child’s LTPA and screen time separately (Table 4 and Table 5). The results show a significant regression equation both for LTPA (F = 5.7970, *p* < 0.001, ΔR2 = 0.2293) and screen time (F = 4.0415, *p* < 0.001, ΔR2 = 0.1577).

For predicting children’s LTPA, the probability values of the father’s education, the father’s occupation status and at least one parent with higher education were less than 0.05. The was a negative correlation between a lower level of father’s education and a longer child’s LTPA, which indicates that a higher level of father’s education is correlated with a higher child’s LTPA. The father’s occupational status (part-time work or not-working) was positively correlated with a longer child’s LTPA.

The father’s occupation status (part-time or not working) correlated positively with a longer screen time. There was a negative correlation between a child’s longer screen time and at least one parent with higher education.

## 4. Discussion

The results of this study extend the current literature by providing a quite detailed description of school-age children’s activities during their leisure time. The importance of PE lessons and the role of in school activities are undeniable. However, the impact of a child’s activities during the after-school period may be remarkable and contribute significantly to the total physical activity level. The percentage of children who were physically active in their free time for at least 1 h each day during the week was about 23%, and those with daily screen time less than 2 h was 33%. This is in line with observations that the majority of children do not meet recommendations for either daily physical activity levels [10,12] or limiting daily screen time [10,25]. Focusing on the after-school period only, Arundell et al. indicated that school-age children spent up to half of their free time in sedentary behaviors [26]. Likewise, Marques et al. reported that television viewing occupied most of the leisure time of boys and girls aged 10–12 years and was followed by computer usage and video game playing [27].

The time of the total child’s physical activity was longer on weekend days, while the average time of sport participation was higher on weekdays. In contrast, the conclusions from systematic literature reviews indicate that school-age children are generally more active on weekdays than on weekend days [19,28,29]. Total screen time was higher on weekend days, and this was observed for both watching TV and playing video games. It seems not surprising that a child’s screen time and physical activity time would be higher on weekend days, because observation time during school days was only a few hours between school and bedtime, while on Saturday and Sunday it lasted all day. However, it is worth emphasizing that the recommendations to reduce screen time to less than 2 h daily are the same for school days and weekend days.

It should be noted that this study evaluated children’s physical activity during their free time only, therefore the actual percentage of children who spend at least 60 min on MVPA would be probably higher if in school physical activities were included. Self-reported data cannot provide as much detailed information as objectively measured physical activity level or sedentary time. Self-reported information may be inconsistent depending on its source (child self-reports or parent proxy reports) [30,31,32]. In this study, the child reported the type and duration of each activity under the supervision of a caregiver, which might potentially minimize bias.

Nonetheless, we observed a positive correlation between a child’s LTPA and parental physical activity level and a negative correlation with a father’s sedentary time. However, no relationship was found between child screen time and parental physical activity or sedentary time. Similar findings obtained by Schoeppe et al. indicate a positive association between maternal and paternal sport participation and children’s leisure time physical activity [33]. This is in line with a systematic review by Petersen et al. according to which the majority of analyzed studies reported a weak positive relationship between parent and child PA [34]. Likewise, Tanaka et al. showed that MVPA in children was positively correlated with maternal MVPA, but there was no significant association between children’s sedentary time or MVPA and paternal MVPA nor parental sedentary time [35]. The study assessing weekday–weekend variations in mother/father–child physical activity, and screen time relationship among families with 5 to 12-year-old children revealed that high levels of parental physical activity contribute to the achievement of the recommended daily physical activity in children on both weekdays and at weekends. Additionally, the excessive weekend sedentary time of parents reduces the odds of the child meeting the recommended daily level of physical activity [36]. According to Hughes et al., for the primary school child-parent dyad, there were medium positive correlations for time sedentary and percentage of the day spend sedentary, but these were statistically non-significant [37]. Jago et al. showed associations between the sedentary time of parents and their daughters [38]. Additionally, higher parental TV viewing was associated with an increased risk of high levels of TV viewing for both boys and girls, but there were no associations between the time that parents and children spend engaged in physical activity [38].

Studies investigating the relationship between parental education and child physical activity or sedentary time remain ambiguous and vary regionally. In general, evidence points to a negative association between parental education and child physical activity in lower economic status countries [39], and a positive relationship between these factors in higher economic status countries [40]. In this study, the multivariate regression model revealed that higher parental education is a predictor of a higher child’s LTPA and shorter screen time, which would suggest that parents with a higher education are presumably more health-conscious. This is in line with the results of the ToyBox study indicating that children with lower maternal, paternal, and parental education levels were less likely to be allocated in the ‘healthy lifestyle’ cluster and more likely to be allocated in the ‘unhealthy lifestyle’ cluster [41].

It is unclear whether sedentary behaviors simply displace physical activity opportunities [42]. Recent studies showed the co-occurrence of different energy-balance-related behaviors among school-age children [43]. Mixed lifestyle patterns were more prevalent than healthy or unhealthy lifestyle patterns [44], with the most frequently observed a mixed physical activity/sedentary behavior pattern, characterized by either high levels of PA with high levels of sedentary behavior or vice-versa [45].

The potential role of schools needs to be underlined. As the family is critical to health behavior change, schools could offer health education and health promotion programs for both children and parents. During preliminary meetings with parents at schools and in consultation with teachers, it was observed that both groups were interested in lifestyle issues, diet, physical activity, and their impact on health. School, as the environment in which children spend a lot of time, might have a great contribution in shaping their health behaviors, screening for unhealthy habits, and stimulating change. However, the collaboration between schools, families, local government and health care professionals needs to improve to increase the efficacy of school-based health promotion interventions [11]. Schools could increase access to PA opportunities, offer after-lessons sports classes, preferably of different disciplines, and promote PA during break and lunch periods. As lower parental education is one of the indicators of lower socioeconomic status, financial barriers to physical activity and sport should also be taken into account, and it could be beneficial for children to attend after-school sports classes that are free of charge, if possible. Healthy lifestyle promotion could include sport, play, or other forms of organized activities that engage both children and parents and promote active forms of spending leisure time. These activities could be an inspiration and help for parents by showing opportunities for how the family can spend free time together and the alternatives to screen time.

### Strengths and Limitations

A possible limitation of this study is the subjectively measured child’s leisure time physical activity and screen time. As the questionnaire focuses only on leisure time physical activities and screen time, other activities during the after-school period, such as doing homework or reading books, were not assessed, although these activities may contribute to total sedentary time. However, this questionnaire was designed to be simple and accessible to fill in by the child for a whole week, and additional questions might change the diary into a more complicated and onerous task, thus resulting in a lower rate of fully completed dairies. The data were collected in autumn (October, November) and spring (March and April). Although there were no observations for wintertime, we cannot exclude the potentially confounding influence of shorter daylight hours, lower temperatures, and rainfall on children’s activity level and screen time. Finally, the cross-sectional design of the study precludes the investigation of casual relationships.

The most important strength of this study is evaluating the familial correlates of early school-age children’s leisure time activities in Poland. This particular age is crucial in promoting and shaping health behaviors, and for screening for low physical activity and excessive screen time. Recent studies suggest that lifestyle intervention effectiveness can be enhanced by including parents [46]. This study also provides evidence that a child’s leisure time is associated with parental education and parental physical activity, which indicates possible health promotion interventions.

## 5. Conclusions

The way that children spend the after-school period may crucially contribute to their total daily physical activity and screen time, however, the percentage of children who comply with the recommendations for a healthy lifestyle is rather low. This study demonstrated that a child’s leisure time activities are associated with parental education and physical activity. Active parents and parents with a higher education tend to have more active children.

These findings indicate the need for healthy lifestyle education among parents of Polish early school-age children and possible directions for health promotion programs. Screening for unhealthy habits, education, guidance, and support for less-educated parents might increase their health consciousness and presumptively result in positive lifestyle changes for the whole family.

## Figures and Tables

**Table 1:** Free-time physical activity during school days and weekend days.

Type of Activity	Mean Time of Activity [min/day]		*p*
	School Days	Weekend	
**Sport**			<0.001
M ± SD	34.13 ± 30.06	19.05 ± 41.61	
Me [Q1; Q3]	27 [12; 48]	0 [0; 21]	
Min–Max	0–176	0–255	
**Active play**			<0.001
M ± SD	47.78 ± 44.16	83.23 ± 91.77	
Me [Q1; Q3]	37 [12; 69]	60 [0; 120]	
Min–Max	0–240	0–420	
**Physical activity (total)**			0.005
M ± SD	89.43 ± 58.12	114.85 ± 99.35	
Me [Q1; Q3]	84 [48; 117]	90 [30; 180]	
Min–Max	0–450	0–420	

**Table 2:** Leisure screen time during school days and weekend days.

Type of Activity	Mean Time of Activity [min/day]		*p*
	School Days	Weekend	
**Watching TV**			<0.001
M ± SD	42.65 ± 41.45	57.82 ± 57.63	
Me [Q1; Q3]	36 [12; 60]	60 [0; 90]	
Min–Max	0–360	0–300	
**Playing computer/tablet etc.**			<0.001
M ± SD	24.18 ± 27.19	38.80 ± 55.08	
Me [Q1; Q3]	18 [0; 36]	30 [0; 60]	
Min–Max	0–120	0–360	
**Screen time (total)**			<0.001
M ± SD	66.10 ± 55.63	95.59 ± 84.69	
Me [Q1; Q3]	55 [30; 96]	90 [30; 135]	
Min–Max	0–430	0–390	

**Table 3:** Spearman’s correlation coefficients (Rho) examining the unadjusted bivariate associations between the mean daily time of a child’s physical activity [minutes/day] and selected parameters of parental physical activity.

Selected Parameters of Parental Physical Activity	Rho	*p*-Value
Mother’s physical activity [MET*min/week]	0.135	0.072
Father’s physical activity [MET*min/week]	0.298	<0.001
Mother’s walking time [min/day]	0.157	0.031
Father’s walking time [min/day]	0.235	0.002
Mother’s sedentary time [min/day]	0.124	0.097
Father’s sedentary time [min/day]	−0.239	0.003

**Table 4:** Results of multivariate regression analysis predicting children’s LTPA using selected parental characteristics.

Variable	B	Std. Err. of B	*β*	*p*-Value
Intercept	64.924	38.292		0.093
Mother’s education	0.159	6.3429	0.0035	0.980
Mother’s occupational status	4.897	7.6139	0.0517	0.521
Father’s education	−27.082	7.3511	−0.593	<0.001
Father’s occupational status	54.070	14.625	0.287	<0.001
Mother’s physical activity level	13.533	8.170	0.170	0.100
Father’s physical activity level	14.538	8.258	0.193	0.081
At least one parent with higher education	41.049	16.621	0.358	0.0149
At least one parent with high PAL *	−18.172	14.953	−0.158	0.227

B—unstandardized coefficient; Std. Err.—standard error; *β*—standardized coefficient; * PAL—physical activity level.

**Table 5:** Results of multivariate regression analysis predicting children’s screen time using selected parental characteristics.

Variable	B	Std. Err. of B	*β*	*p*-Value
Intercept	103.089	35.670		0.002
Mother’s education	−6.770	5.897	−0.168	0.144
Mother’s occupational status	−3.797	7.101	−0.045	0.551
Father’s education	−6.672	6.815	−0.163	0.480
Father’s occupational status	43.290	13.630	0.257	0.002
Mother’s physical activity level	−4.950	7.607	−0.069	0.505
Father’s physical activity level	−1.301	7.688	−0.019	0.754
At least one parent with higher education	−61.566	15.431	−0.602	<0.001
At least one parent with high PAL *	−0.357	13.854	−0.003	0.993

B—unstandardized coefficient; Std. Err.—standard error; *β*—standardized coefficient; * PAL—physical activity level.4.

## Data Availability

The data presented in this study are available on request from the corresponding author.
